# The use of a sternothyroid muscle flap to prevent the re-recurrence of a recurrent tracheoesophageal fistula found 10 years after the primary repair

**DOI:** 10.1186/s40792-016-0213-y

**Published:** 2016-09-02

**Authors:** Hajime Takayasu, Kouji Masumoto, Miki Ishikawa, Takato Sasaki, Kentaro Ono

**Affiliations:** Department of Pediatric Surgery, Faculty of Medicine, University of Tsukuba, 1-1-1, Tennoudai, Tsukuba, Ibaraki 305-8575 Japan

**Keywords:** Esophageal atresia, Tracheoesophageal fistula, Recurrence, Sternothyroid muscle flap, Hiatus hernia

## Abstract

Recurrent tracheoesophageal fistula (TEF) is still difficult to diagnose and repair. In almost all cases, recurrence appears relatively soon after the primary surgery. We herein describe a case of recurrent TEF that appeared 10 years after the primary repair. At 2 years of age, the patient suffered from mental retardation due to encephalitis and developed a hiatus hernia with gastro-esophageal reflux. He underwent the repair of a hiatus hernia and fundoplication at 3 years of age. However, the hiatus hernia recurred 6 months after the operation. The patient suffered from recurrent pneumonia for 6 years after the appearance of the recurrent hiatus hernia. At 9 years of age, he was hospitalized frequently due to recurrent severe pneumonia. After admission at 9 years of age, an endoscopic study under general anesthesia was performed and revealed subglottic stenosis and a dilated esophagus with a recurrent hiatus hernia. Tracheotomy or laryngotracheal separation was first planned in order to improve his upper airway and facilitate the safer repair of the recurrent hiatus hernia. After laryngotracheal separation, the patient still suffered from severe pneumonia. In addition, a small volume of nutritional supplement was aspirated from the tracheostomy. Thus, recurrent TEF was suspected. Tests using dye under both esophagoscopy and bronchoscopy confirmed recurrent TEF. The fistula recurred in the cervical area because of the elevation of the esophagus due to the recurrent hiatus hernia. The fistula was surgically closed, with a sternothyroid muscle flap to prevent re-recurrence. At 4 months after this operation, the recurrent hiatus hernia was repaired. Thereafter, the patient’s respiratory symptoms showed a dramatic improvement. The patient is now doing well and free from further recurrences of TEF and hiatus hernia at 2 years after the final operation.

## Background

Previous studies involving large series of patients have revealed that recurrent tracheoesophageal fistulas (TEFs) occur in 6–16 % of patients who undergo esophageal atresia (EA)/TEF repair, typically in the first year of life [1–8].

Various factors are implicated in the formation of recurrent TEFs. Recurrence is more likely to occur in patients with anastomotic leakage [3, 5, 6, 8]. Esophageal perforation during the dilatation of an anastomotic stricture is another risk factor for the recurrence of TEF [5, 6, 8]. In addition, when magnified by poor esophageal peristalsis, gastro-esophageal reflux (GER) may contribute to the recurrence of TEF, because it causes the upper esophagus to have prolonged acid contact.

Reoperation for recurrent TEF usually includes the interposition of adjacent soft tissue [5, 6]. Several authors have reported the effects of the interposition of viable tissue, as a flap, between the suture lines of the trachea and esophagus to prevent further recurrence in patients with recurrent TEF [9, 10]. We experienced a rare case in which a TEF recurred in the cervical area on the 10th year after the primary repair. In this case, we chose the sternothyroid muscle flap because it was easy to harvest and because it has been shown to be reliable in the repair of fistulas that recur in the cervical area. Based on our experience of this case, we reviewed and discuss the pathogenesis and the diagnosis of the recurrent TEF and the effectiveness of the sternothyroid muscle flap for preventing further recurrence.

## Case presentation

A 9-year-old boy had presented with frequent pneumonia. He was born vaginally at 25 weeks and 6 days at home due to an unknown reason. His birth weight was 750 g. He was in cardiopulmonary arrest when he arrived at the previous hospital. Resuscitation was performed immediately on arrival. Peritoneal drainage was performed because gastric perforation was found soon after resuscitation. Thereafter, a diagnosis of TEF was made, and gastrostomy was created in the previous hospital. He was transferred to our institution at 28 days of age. He was diagnosed as having an EA with a distal TEF (Gross C), and the primary repair of the EA with fistula division was performed at our hospital when he was 5 months of age. The postoperative course was uneventful, and gastrostomy was closed at 10 months of age. He discharged at 2 year of age. After his discharge, severe epilepsy and encephalopathy due to encephalitis occurred, and he became disabled with severe mental retardation at the age of 2. He could not feed orally. At the age of 3, he underwent Nissen fundoplication and re-gastrostomy because of gastroesophageal reflux and hiatus hernia. At the time of the operation, subglottic stenosis was suspected because he could only be intubated with a tracheal tube of 2.5 mm in diameter.

Six months after fundoplication, a recurrence of hiatus hernia was found, which involved the herniation of the stomach and small and large intestine into the thoracic cavity. A reoperation to repair the herniation was not performed because the patient had been in stable condition. However, he had been suffering from recurrent respiratory tract infections since he was 7 years of age. In addition, severe bronchial asthma worsened his respiratory symptoms. At 9 years of age, he was referred to our hospital again because of frequent pneumonia.

**Fig. 1:**
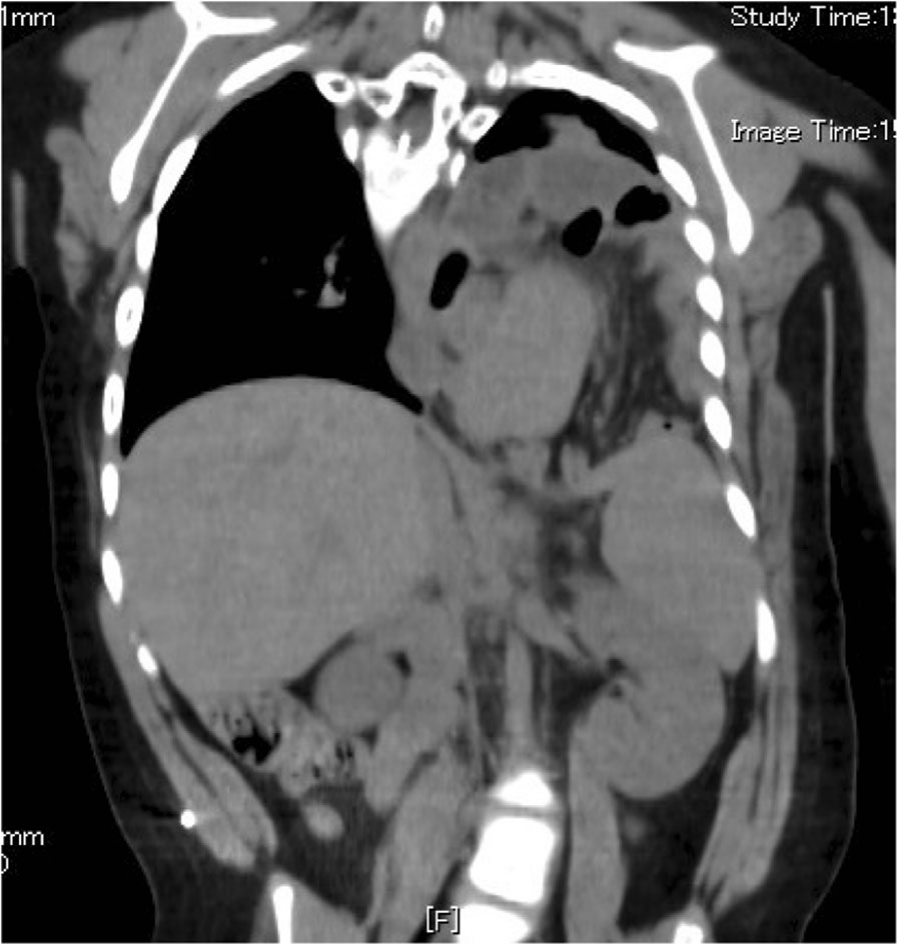
A CT scan revealed the herniation of the stomach and the large and small intestine into the thoracic cavity

A CT scan revealed the herniation of the large and small intestine into the thoracic cavity; we were therefore of the opinion that they should be pulled down into the abdominal cavity in order to resolve the patient’s respiratory problems (Fig. 1). We thought that we should perform tracheotomy or laryngotracheal separation before repairing the recurrent hiatus hernia because the patient presented with severe dysphagia as well as severe hiatus hernia. An upper gastrointestinal series and endoscopy were performed to evaluate the esophagus and stomach, revealing the presence of residual gastroesophageal reflux and an elevated esophagus due to the recurrent hiatus hernia.

**Fig. 2:**
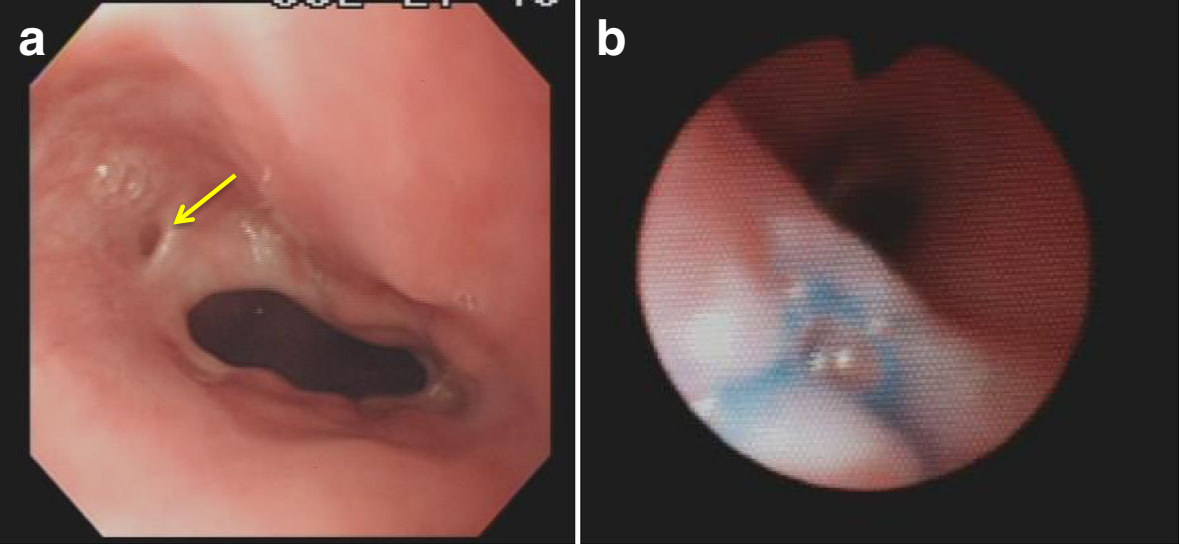
Esophagoscopy showed a pit (*arrow*) beside the anastomotic line (**a**). Indigo carmine, which had been injected via a catheter placed in the pit appeared with an air bubble from the posterior wall of trachea (**b**). Thus, the patient was diagnosed with recurrent TEF

At 10 years of age, tracheotomy was performed. However, the patient’s respiratory status did not improve; thus, we performed laryngotracheal separation 6 weeks later. Three weeks after the laryngotracheal separation, a small volume of nutritional supplement, which had been administered via gastrostomy, was aspirated from the tracheotomy. We therefore suspected the recurrence of TEF. Under general anesthesia, both esophagography and endoscopy were performed again. The anastomotic line of the esophagus was located in the cervical area, 15 cm from the incisors. A pit was identified just beside the anastomotic line (Fig. 2a). Indigo carmine injected from the catheter placed in the pit revealed that the recurrent fistula was located in the trachea (Fig. 2b). In addition, the anastomosis line of EA was thought to have been elevated to the cervical area due to the recurrence of the hiatus hernia.

**Fig. 3:**
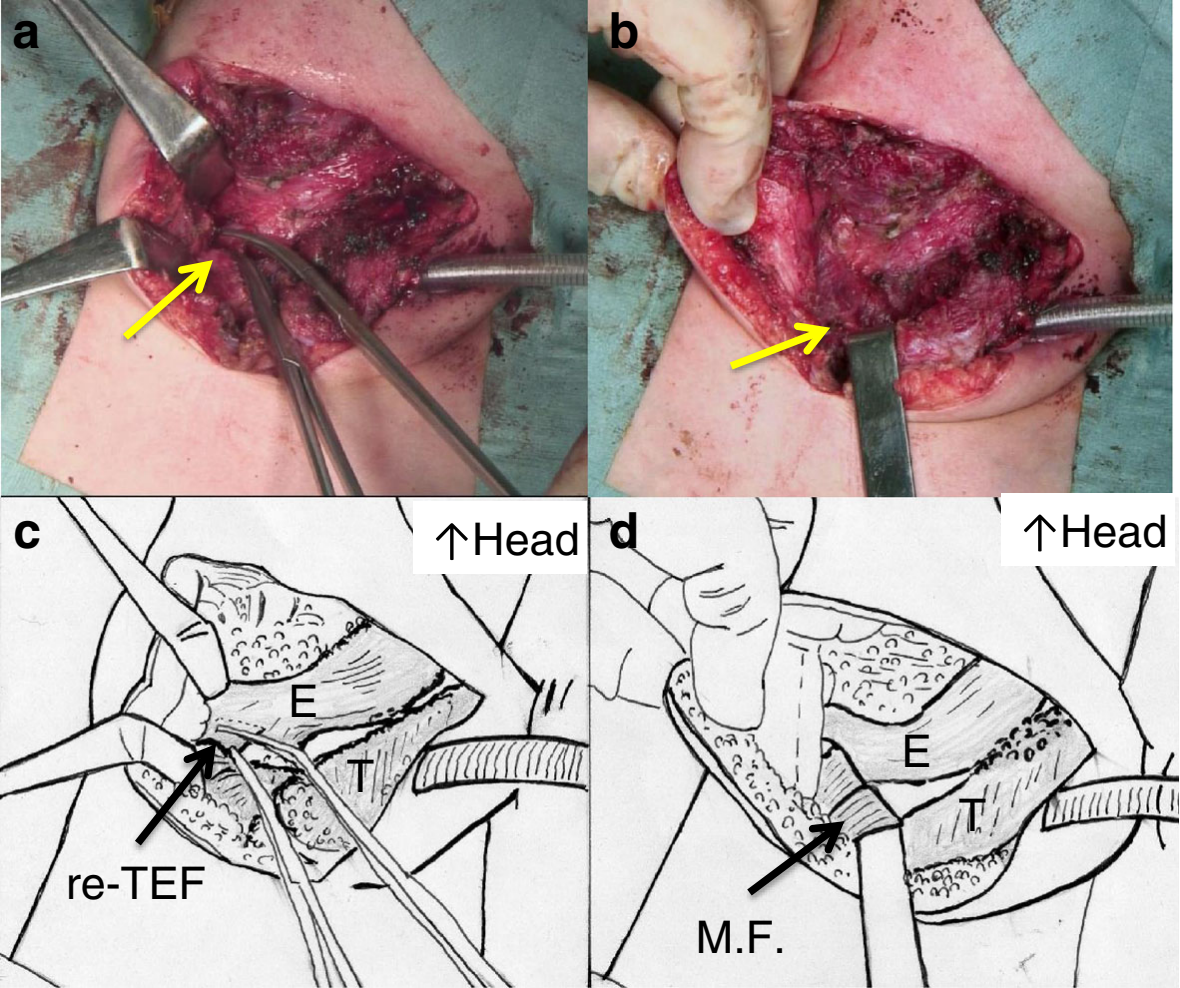
**a** The esophagus and trachea was sharply divided, and the recurrent TEF was resected. A Pean’s forceps was used to grasp both ends of the fistula (*arrow*). The fistula orifice in the esophagus was repaired with interrupted suture. The tracheal wall was also sutured. **b** A sternothyroid muscle flap was directly sutured to the esophagus (*arrow*) and interposed between the esophagus and the trachea. **c**, **d** The protocol of **a** and **b**, respectively. *E* esophagus, *T* trachea, *re-TEF* recurrent tracheoesophageal fistula, *M.F.* sternothyroid muscle flap

Thus, the repair of the recurrent TEF was performed through the right cervical approach. It was impossible to place a catheter or guide wire through the TEF. After the separation of the esophagus and trachea, the small hole of the fistula was found and closed (Fig. 3a, c). At the site of the fistula, the esophagus and trachea had a “common wall” due to severe adhesion around the fistula. A sternothyroid muscle flap was interposed between the closure sites (Fig. 3b, d). After the operation, the respiratory symptoms of the patient improved dramatically. Three months after the repair of the recurrent TEF, the recurrent hiatus hernia was surgically treated. The postoperative course was also uneventful. The patient remains well at 2 years after the surgical repair of the recurrent TEF. There is no evidence of the further recurrence of TEF or hiatus hernia.

### Discussion

We experienced a relatively rare case of recurrent TEF that was found 10 years after the initial repair. We herein discuss the reason why the recurrent TEF had not been identified and the surgical technique that we used to repair the patient’s recurrent TEF.

The patient had subglottic stenosis, which made it difficult to observe the trachea. In addition to the subglottic stenosis, both bronchial asthma and recurrent hiatus hernia were thought to have caused the patient’s respiratory symptoms due to frequent pneumonia. These factors may have caused the patient’s recurrent TEF to remain unidentified. Recurrence was first suspected when a small volume of nutritional supplement that had been administered via gastrostomy was aspirated from the tracheostomy tube after the laryngotracheal separation. In general, although many cases of recurrent TEF are diagnosed by a contrast study, some require further examination by endoscopy with or without a dye test [5, 6, 8, 11]. In the present case, bronchoscopy (which is safer and precise) was not possible prior to tracheostomy due to the patient’s subglottic stenosis. As shown in Fig. 2, esophagoscopy and bronchoscopy combined with a dye test enabled us to diagnose the recurrent TEF.

In general, almost all patients with recurrent TEF have episodes of leakage and/or tracheal injuries [4–6, 8]. The recurrent fistula usually opens in the trachea at the site of the original fistula site or tracheal injury [4–6]. In this case, there was no leakage or tracheal injury at the site of the primary repair. In contrast with other cases, the site of recurrence was found to be high in the cervical area. Thus, we suspected that the patient had both proximal and distal tracheoesophageal fistula (gross type D), and the upper pouch fistula was missed in the primary operation [12, 13]. However, as shown in Fig. 2, a pit was identified just beside the anastomotic line. The anastomotic line in the esophagus had shifted to the cervical area because of the recurrent hiatus hernia. We therefore judged that the original fistula (proximal tracheoesophageal fistula of gross type D) in the upper pouch was not found at this time. We also suspected that the trachea had been injured during laryngotracheal separation. However, during the repair of the recurrent TEF, the fistula was found to be located in an area that had not been touched during the laryngotracheal separation. Based on the above-noted findings, we diagnosed the patient with recurrent TEF.

In the present case, the TEF might have recurred at the age of 9, when the patient started suffering from frequent and severe pneumonia. The recurrent hiatus hernia caused GER and the poor clearance of refluxed gastric juice in the esophagus. Prolonged acid contact causing inflammation and erosion around the anastomotic site is thought to have been important in the pathogenesis of TEF in the present case.

There are several options for repairing recurrent TEF. Endoscopic techniques using adhesives have been presented [14–16]. This method is reported to be effective when the fistula tract is first de-epithelialized using several types of devices and then fibrin glue or a similar agent is injected into the tract of the fistula [5, 14–16]. However, it is thought that this technique would have been difficult in the present case because the tract was not detected clearly and because it was not possible to place a catheter under endoscopy. We therefore opted for open surgical closure. The placement of viable tissue between the suture lines may help to prevent the further recurrence of TEF [5, 6, 9, 10]. Previous studies have described the use of the pericardium, pleura, intercostal flap, cervical muscle flap, cartilage, and lymph nodes for this purpose [5, 6, 9, 10]. The flap can be harvested from the same surgical site with its own blood supply. In the present case, a sternothyroid muscle flap was chosen because the recurrent fistula was in the cervical area. In fact, in the present case, the muscle flap was easily and safely interposed between the divided ends of the fistula with a good blood supply. Based on our experience, the interposition of a muscle flap using the sternothyroid muscle is thought to be a good option for reoperation to repair a recurrent TEF in the cervical area.

## Conclusions

In summary, we described a case of recurrent TEF that was found 10 years after the initial surgery. The recurrent fistula was located in the cervical area, which was an unusual area for a TEF to recur. It is important to suspect recurrent TEF when a patient repeatedly suffers from respiratory infections because TEFs may recur long after the primary repair, as we reported in our case. The definitive diagnosis of recurrent TEF, which was made by dye test under endoscopy, helped us to perform an accurate repair. In addition, as shown in this report, the usage of a sternothyroid muscle flap as an interposition material between the esophagus and trachea was thought to have been an effective approach for preventing the further recurrence of TEF.

## References

[1] Pinheiro PF, Simões e Silva AC, Pereira RM (2012). Current knowledge on esophageal atresia. World J Gastroenterol.

[2] Castilloux J, Noble AJ, Faure C (2010). Risk factors for short- and long-term morbidity in children with esophageal atresia. J Pediatr.

[3] Zhang Z, Huang Y, Su P, Wang D, Wang L (2010). Experience in treating congenital esophageal atresia in China. J Pediatr Surg.

[4] Kovesi T, Rubin S (2004). Long-term complications of congenital esophageal atresia and/or tracheoesophageal fistula. Chest.

[5] Bruch SW, Hirschl RB, Coran AG (2010). The diagnosis and management of recurrent tracheoesophageal fistulas. J Pediatr Surg.

[6] Coran AG (2013). Redo esophageal surgery: the diagnosis and management of recurrent tracheoesophageal fistula. Pediatr Surg Int.

[7] Spitz L (2006). Esophageal atresia. Lessons I have learned in a 40-year experience. J Pediatr Surg.

[8] Koivusalo AI, Pakarinen MP, Lindahl HG, Rintala RJ (2015). Revisional surgery for recurrent tracheoesophageal fistula and anastomotic complications after repair of esophageal atresia in 258 infants. J Pediatr Surg.

[9] Sugiyama A, Urushihara N, Fukumoto K, Fukuzawa H, Watanabe K, Mitsunaga M (2013). Combined free autologous auricular cartilage and fascia lata graft repair for a recurrent tracheoesophageal fistula. Pediatr Surg Int.

[10] Chavin K, Field G, Chandler J, Tagge E, Othersen HB (1996). Save the child’s esophagus: management of major disruption after repair of esophageal atresia. J Pediatr Surg.

[11] Zhu H, Shen C, Xiao X, Dong K, Zheng S (2015). Reoperation for anastomotic complications of esophageal atresia and tracheoesophageal fistula. J Pediatr Surg.

[12] Patel RV, Greene O, Motiwale S, Singh S. The combination of pure oesophageal atresia with an associated missed H-type tracheo-oesophageal fistula. BMJ Case Rep. 2013 Jul 29;2013. pii: bcr2013200198. doi: 10.1136/bcr-2013-200198. 10.1136/bcr-2013-200198 PMC3736222 23897389

[13] Guo W, Li Y, Jiao A, Peng Y, Hou D, Chen Y (2010). Tracheoesophageal fistula after primary repair of type C esophageal atresia in the neonatal period: recurrent or missed second congenital fistula. J Pediatr Surg.

[14] Gutiérrez San Román C, Barrios JE, Lluna J, Ibañez V, Hernández E, Ayuso L, Valdes E (2006). Long-term assessment of the treatment of recurrent tracheoesophageal fistula with fibrin glue associated with diathermy. J Pediatr Surg.

[15] Richter GT, Ryckman F, Brown RL, Rutter MJ (2008). Endoscopic management of recurrent tracheoesophageal fistula. J Pediatr Surg.

[16] Meier JD, Sulman CG, Almond PS, Holinger LD (2007). Endoscopic management of recurrent congenital tracheoesophageal fistula: a review of techniques and results. Int J Pediatr Otorhinolaryngol.

